# Pre-diagnostic cruciferous vegetables intake and lung cancer survival among Chinese women

**DOI:** 10.1038/srep10306

**Published:** 2015-05-19

**Authors:** Qi-Jun Wu, Gong Yang, Wei Zheng, Hong-Lan Li, Jing Gao, Jing Wang, Yu-Tang Gao, Xiao-Ou Shu, Yong-Bing Xiang

**Affiliations:** 1 State Key Laboratory of Oncogene and Related Genes, Shanghai Cancer Institute, Renji Hospital, Shanghai Jiaotong University School of Medicine, Shanghai, China; 2 Department of Epidemiology, Shanghai Cancer Institute, Renji Hospital, Shanghai Jiaotong University School of Medicine, Shanghai, China; 3 Division of Clinical Epidemiology, Shengjing Hospital of China Medical University, Shenyang, China; 4 Division of Epidemiology, Department of Medicine, Vanderbilt Epidemiology Center, Vanderbilt-Ingram Cancer Center, Vanderbilt University School of Medicine, Nashville, TN, USA

## Abstract

No study to date has prospectively evaluated the association between pre-diagnostic cruciferous vegetables intake and lung cancer survival among women. This analysis included 547 incident lung cancer cases identified from the Shanghai Women’s Health Study (SWHS) during the follow-up period of 1997-2011. Dietary intake was assessed for all SWHS participants at enrollment and reassessed 2-3 years later. Cox proportional hazards models were used to estimate hazard ratios (HRs) and 95% confidence intervals (CIs) with adjustment for potential confounders. Of the 547 lung cancer patients, 412 patients died during the follow-up. A total of 393 (95.4%) deaths from lung cancer were documented with median survival time of 10.3 months (interquartile range, 3.6-21.1 months). High cruciferous vegetables intake was significantly associated with improved lung cancer-specific survival after adjusting for all nonclinical prognostic factors (n = 547, HR = 0.69; 95%CI = 0.49-0.95; *P* trend = 0.02) for the highest *versus* lowest quartile. A slightly stronger association of cruciferous vegetables intake with lung cancer-specific survival was observed in analyses restricted to patients with known clinical prognostic factors (n = 331, HR = 0.63; 95%CI = 0.41-0.97; *P* trend = 0.03) or never smokers (n = 308, HR = 0.58; 95%CI = 0.37-0.91; *P* trend = 0.02). In conclusion, pre-diagnostic cruciferous vegetables intake is associated with better survival of lung cancer in Chinese women.

Lung cancer is the second leading cause of cancer death among women with approximately 0.5 million deaths worldwide in 2012^1^. It is estimated that lung cancer deaths accounted for 22.6% of all cancer deaths among Chinese women in 2012^2^. Most lung cancer patients are diagnosed at an advanced disease stage, and thus the prognosis for this cancer is very poor, with a 5-year survival rate of only 15%^3^. Since the high mortality of this disease and early detection which continues to be an elusive goal^3^, the investigation into modifiable risk factors of survival of lung cancer, specifically diet, has received increasing attention.

Cruciferous vegetables have been of specific interest mainly due to their contents, indoles and isothiocyanates (ITCs), which are derived from glucosinolate precursors by the action of myrosinases, might hinder lung tumorigenesis through inhibition of tobacco-specific pro-carcinogens by phase I enzymes (e.g. Cytochrome P-450s) and enhancement of detoxification by phase II enzymes^4^,^5^. Besides, *in vitro* and *in vivo* experiments indicated that a number of ITCs may have several antitumor properties such as inducing apoptosis and oxidative stress^6^,^7^,^8^,^9^, inhibition of angiogenesis^8^,^9^,^10^,^11^, metastasis^10^,^11^, and cell cycle progression^8^,^9^,^12^,^13^.

Our recent study from the Shanghai Women’s Health Study (SWHS) demonstrated that cruciferous vegetables intake significantly decreased 41% incident lung cancer risk among female nonsmokers^14^. Although several meta-analyses have also confirmed the protective role of cruciferous vegetables intake in lung cancer risk^14^,^15^, no study to date has focused on the association between pre-diagnostic cruciferous vegetables intake and the lung cancer survival. Herein, in this report, we prospectively investigate the hypothesis that women with lung cancer with higher pre-diagnostic cruciferous vegetables may have better prognosis.

## Results

The mean ages of the study participants were 59.2 years (standard deviation [SD], 8.5 years) at baseline interview, and 66.7 years (SD, 9.0 years) at lung cancer diagnosis. The median intervals since the first and the second questionnaires among all lung cancer patients (n = 547) were 7.9 years (interquartile range, 4.7-10.4 years) and 5.8 years (interquartile range, 2.7-8.0 years). The median intervals since cancer diagnosis among all lung cancer patients (n = 547) and censored patients (n = 135) were 13.7 month (interquartile range, 5.1-29.8 months) and 34 months (interquartile range, 19-61 months), respectively. A total of 412 deaths were documented during the follow-up with median survival time of 10.3 months (interquartile range, 3.6-21.1 months), including 393 deaths (95.4%) from lung cancer and 19 deaths (4.6%) due to other causes. Detailed selected age-adjusted demographic characteristics, lifestyle habits, and clinical information by quartiles of cruciferous vegetables intake are presented in Table 1. Women who had high levels of cruciferous vegetables intake tended to also have higher BMI, intake of more total energy, fruits, soy foods, and non-cruciferous vegetables but were less likely to smoke cigarette. The table 2 summarizes selected patients’ characteristics in relation to lung cancer specific survival after mutual adjustment for each other. Older age at diagnosis, ever exposure to cigarette smoking, and more advanced tumor stage were significantly associated with lung cancer-specific survival in the multivariate models. In contrast, ever exposure to tea drinking was significantly inversely associated with lung cancer-specific survival (Table. 2). Of note, the results of ever have chemotherapy and surgery showed borderline significance which indicated that following chemotherapy and surgery after lung cancer diagnosis may improve the survival of these patients.

**Figure 1:**
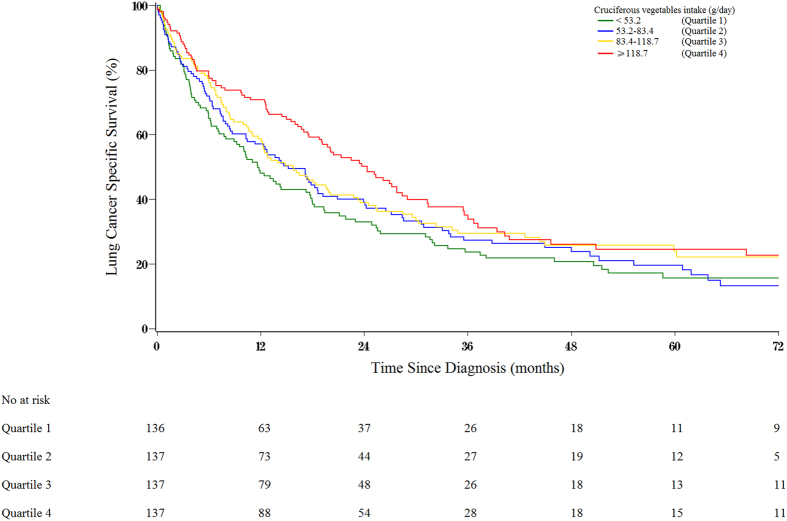
Multivariable-adjusted lung cancer-specific survival curves of patients (n = 547) with lung cancer by pre-diagnostic intake of cruciferous vegetables, estimated from a stratified proportional hazards model (stratified on birth year and adjusted for age at diagnosis, body mass index, tea drinking, cigarette smoking, intakes of energy, fruit and non-cruciferous vegetables) by using a direct adjustment method^36^. The green line indicates the Q1 (lowest) of intake, the blue line indicates the Q2, the gold line indicates the Q3, and the red line indicates the Q4 of intake (highest).

The results of analyses for total and subsets of patients for pre-diagnostic cruciferous vegetables intake and lung cancer-specific survival are listed in Table. 3. In the analyses of all patients, higher cruciferous vegetables intake was associated with statistically significant better lung cancer-specific survival. The HR comparing the highest and the lowest quartiles of cruciferous vegetables intake were 0.69 (95% CI = 0.49-0.95, *P* trend = 0.02) for lung cancer-specific survival after adjusting for demographic and lifestyle characteristics and other nonclinical prognostic factors (Table 3). Fig. 1 visually depicts the association between cruciferous vegetables intake and lung cancer-specific survival among all patients. Compared with the lowest quartiles of cruciferous vegetables intake, survival was higher in patients with the highest quartiles of intake (log rank, *P* = 0.01).

The inverse association of cruciferous vegetables intake became slightly stronger when the analysis was restricted to 331 patients with data available on known clinical prognostic factors and adjustment for tumor stage and treatment, with the corresponding HR = 0.63 (95% CI = 0.41-0.97; *P* trend = 0.03) and to 308 nonsmoking patients, with the corresponding HR = 0.58 (95% CI = 0.37-0.91; *P* trend = 0.02). We also conducted the nonlinear dose-response analysis among patients with known clinical prognostic factors (n = 331), but there was no evidence of a nonlinear association between cruciferous vegetables intake and lung cancer-specific survival (*P* nonlinearity = 0.32).

In sensitivity analyses, after excluding the cases (n = 31) who were diagnosed within the first year after baseline and the first follow-up FFQ, we still observed inverse associations between pre-diagnostic cruciferous vegetables intake and lung cancer-specific survival among different subsets of patients which were similar to the main results (Supplementary Table 1). However, the trend test of lung cancer-specific survival was not statistically significant among patients with data available on known clinical prognostic factors (*P* trend = 0.16). Furthermore, similarly, the results were robust when using cumulative averaging method^16^ to update the dietary exposures (Supplementary Table 2) or only the baseline FFQ to assess the dietary exposures (Supplementary Table 3).

## Discussion

To our knowledge, this is the first investigation reporting a survival advantage among women with lung cancer who intake higher pre-diagnostic cruciferous vegetables. Furthermore, the associations appeared stronger among patient with known clinical characteristics or nonsmoking women (Table 3). These stated findings, coupled with our previous observation of an approximately 27% reduction in risk of incident lung cancer associated with high cruciferous vegetables intake and 41% reduction for the female nonsmokers^14^, provides further evidence for a potential protective role of cruciferous vegetables intake in lung cancer development and prognosis.

The current epidemiologic evidence of cruciferous vegetables intake on total/lung cancer specific mortality or survival were sparse^17^,^18^,^19^,^20^, and until recently, there has been no prior epidemiologic study report on the association between cruciferous vegetables intake and survival among lung cancer patients. This study adds to the limited evidence on the role of diet and survival among individuals diagnosed with lung cancer. A previous study which based on the SWHS, Zhang *et al.*^17^ have found that cruciferous vegetables intake was associated with a significantly reduced risk of overall and cardiovascular mortality but failed to provide support for a protective effect on overall cancer mortality which suggested that the association between cruciferous vegetables intake and lung cancer might be specific.

As the first study pointed to the beneficial effect of cruciferous vegetables intake on lung cancer patients, though the exact biologic mechanisms underlying the aforementioned association are not fully understood, the protective role of cruciferous vegetables intake is biologically plausible on the basis of *in vivo* and *in vitro* data. ITCs, occurring as the hydrolytic product of glucosinolates in cruciferous vegetables^21^ have antiproliferative, antimetastasis, and antiangiogenesis activities^8^,^9^,^11^,^22^,^23^. Additionally, the potent activity of induction of apoptosis by ITCs induced highly metastatic lung cancer cell apoptosis in a dose-dependent manner and caused cell cycle arrest at the G_2_/M phase^6^,^24^, as well as gefitinib-resistant non-small cell lung cancer^25^. Furthermore, *in vivo* studies suggested that several specific ITCs (e.g., benzyl ITC (BITC) and phenethyl ITC (PEITC)) were not only linked with activation of activator protein-1 (AP-1), which was one of the downstream targets of mitogen-activated protein kinase (MAPK), and induction of apoptosis in lung tissues but inhibited malignant progression to lung adenocarcinomas^26^,^27^. Since most patients present with locally advanced (37%) or metastatic (38%) disease at the time of diagnosis and metastasis is the most common cause of death in lung cancer patients^28^, several *in vitro* studies not only found that BITC and PEITC suppressed lung cancer cell metastasis potential by down-regulation the expression of transcription factor twist and increasing the expression of β-catenin which played an essential role in cancer invasion and metastasis but inhibited both Akt phosphorylation and NF-κB transcriptional activation in a dose-dependent manner which is a major anti-apoptotic/pro-survival pathway^6^,^12^. A recent study using 1005 middle-aged Chinese women within the SWHS have reported that higher intakes of cruciferous vegetables or their constituents are associated with lower the concentrations of several inflammatory and oxidative stress markers including tumor necrosis factor-α, interlukin-1β (IL-1β), and IL-6^29^.

Since the possibility that patients with lung cancer may have changed their dietary intake, to minimize this bias, we carried out the sensitivity analysis excluding lung cancer cases within first year of baseline and first follow-up FFQ completion (Supplementary Table S1). Additionally, similar results were observed when using cumulative averaging method^30^ or only the baseline FFQ to assess the dietary exposures (Supplementary Table 2 and Supplementary Table 3). Noteworthy, we also carried out the analyses of the relationship between non-cruciferous vegetables intake and survival of lung cancer but found null results (data not shown), which support a special association for cruciferous vegetables intake.

Several key strengthens of this study should be addressed. First, the SWHS is a prospective study with high participation rates for baseline survey and retention rates for follow-up, which minimize the potential differential recall bias or selection bias. Additionally, dietary information was assessed by an in-person interview with a validated FFQ over two time periods, which provided more stable estimates of usual intake of cruciferous vegetables. Second, a large sample size with low cigarette smoking rate (8.7%) of the patients included in this study not only made it possible to consider a wide range of potential confounders in the multivariable models but minimize the potential confounding effects due to cigarette smoking. Third, Asian populations are known to habitually consume large amounts of cruciferous vegetables and other plant-based foods, which provides a unique opportunity to address hypotheses related to the potential health properties of these foods^29^.

Despite the clear strengths of this study, we also acknowledge several limitations. Firstly, the problem of missing data might be a concern. In our study, 39.8% of patients had missing tumor stage information, which may limit the generalizability of our results of cruciferous vegetables intake to all lung cancer patients. However, slight differences in the association was observed in the analyses with and without adjustment for clinical prognostic factors (Table. 3) and little evidence showed that these known clinical factors were associated with cruciferous vegetables intake (Table. 1). Moreover, we found no difference of nonclinical prognostic factors between these patients with and without the tumor stage information (data not shown). Second, we cannot rule out that our findings may have been influenced in part by residual confounding, resulting from measurement errors or unmeasured lifestyle factors. Nonetheless, we carefully adjusted for possible confounding covariates in multivariate models without observing meaningful changes in risk estimates. Although previous study has reported that pre-diagnostic soy food intake was favorably affect the all-cause survival of patients with lung cancer^31^, the low correlation between cruciferous vegetables and soy food intake (r = 0.27) and the results were similar in the multivariable models with and without adjustment for soy food intake (data not shown), which also suggested that cruciferous vegetables intake may independently improve survival of lung cancer in women. Finally, we were unable to investigate the association between post-diagnosis cruciferous vegetables intake and lung cancer survival in this study because the post-diagnosis dietary intake information was not available for most of the included patients which was attributed to the poor prognosis and short survival period of this disease.

In conclusion, our findings indicate that higher pre-diagnostic cruciferous vegetables intake was associated with better lung cancer-specific survival of lung cancer patients in women, particularly among nonsmokers. Considering our previous findings of reduced lung cancer risk with increasing intake of cruciferous vegetables in women^14^, the association observed in this study that higher intake of cruciferous vegetables before diagnosis of lung cancer may have beneficial impact on patient’s clinical outcomes further reinforce the importance of cruciferous vegetables consumption among women. Future investigations are warranted to confirm our findings and find whether this association differs by the specific types of cruciferous vegetables, histologic type, and tumor stage.

## Methods

### Study Participants

From 1997 to 2011, a total of 585 incident cases of lung cancer (International Classification of Diseases, 9th Revision; codes 162.0-162.9) were documented in the participants of the SWHS, which is a population-based, prospective cohort study conducted in urban Shanghai, China. Details of the study design and methods of the SWHS have been published previously^32^. Briefly, the SWHS recruited 74,941 females aged 40-70 years from 1997 to 2000, residing in seven urban communities of Shanghai, with a response rate of 92.7%. Cohort members were followed for occurrence of cancer by in-person follow-up surveys every 2-3 years and annual record linkage with databases from the population-based Shanghai Cancer Registry with the average follow-up response rate of 96.8%. All possible cancer diagnoses were verified through home visits. The SWHS was approved by the Institutional Review Boards for human research from the Vanderbilt University and National Cancer Institute of United States and Shanghai Cancer Institute. All participants provided written informed consent. Our study was carried out in accordance with the approved guidelines. We excluded patients with missing information on cancer treatment (n = 29) and patients with the same date of death and cancer diagnosis (n = 9). Thus, during a median follow-up of 13.2 years (interquartile range, 12.4-13.8 years), a total of 547 incident patients with lung cancer were included in the final analysis.

### Data Collection

#### Measurement of pre-diagnostic dietary intake

Dietary information was collected using a validated semi-quantitative food-frequency questionnaire (FFQ) at baseline and the first follow-up conducted 2-3 years after baseline^32^,^33^. The FFQ included 77 food items, which included about 90% of commonly consumed food items in urban Shanghai in 1996^32^. Five cruciferous vegetables (5 items) commonly consumed in this population were included in the questionnaire, including Chinese greens (bok choy), green cabbage, Chinese cabbage, cauliflower, and white turnip/radish. Among these, Chinese greens (bok choy) are the most likely consumed cruciferous vegetables in the populations of the SWHS^14^. This FFQ was validated by multiple 24-hour dietary recalls in a subset of participants of the SWHS (n = 200)^33^. The Pearson correlation coefficients between self-reported intakes from the FFQ and these multiple 24-hour dietary recalls were 0.41 for all vegetables and 0.55 for fruits. Total energy intakes were calculated on the basis of the Chinese Food Composition Tables^34^.

To improve estimates for usual pre-diagnostic dietary intake, we used the average intakes estimated from the first FFQ (at baseline) and the second FFQ (conducted 2-3 years after baseline) surveys for 474 (86.7%) of the 547 patients included in this study. For patients who provided no second FFQ data or reported having chronic diseases (e.g., diabetes, cardiovascular) or lung cancer diagnosed between the two FFQs, only the intake estimates from the first FFQ were used (13.3%)^31^.

#### Study Outcomes

Survival time began on the date of lung cancer diagnosis and ended on the date of death or the date of latest linkage to Shanghai Vital Statistics Registry (December 31, 2011), whichever came first.

Since over 95% of patients (393 in 412) died from lung cancer in this study, therefore, the primary outcome is lung cancer-specific survival which is defined as time from the date of lung cancer diagnosis to death from lung cancer. Cause of death was obtained from the death certificates.

#### Covariates

Information on demographic characteristics, anthropometric measurements, lifestyle habits, medical history, passive smoking, and other exposures was obtained through the baseline survey. Clinical information, including tumor stage, histological type, and treatment (surgery, chemotherapy, or radiotherapy), was collected by medical chart review by health professionals (registered nurses or physicians) and the information was verified by a senior oncologist. Tumor stage was defined according to criteria of the American Joint Committee on Cancer (seventh edition)^35^.

#### Statistical Analysis

Differences in demographic and clinical characteristics across cruciferous vegetables intake categories were evaluated using the Kruskal-Wallis test for continuous variables and *χ*^2^ test for categorical variables. Cox proportional hazards models with age as time scale were used to calculate hazard ratios (HRs) and 95% confidence intervals (CIs). Cruciferous vegetables intakes were categorized by quartile distribution, with the lowest quartile serving as the reference group. Tests for linear trend were performed by assigning the median value of consumption for each quartile of cruciferous vegetables and treating it as a continuous variable in the regression model. A direct adjustment method on the basis of a stratified proportional hazards model was used to estimate covariate-adjusted survival curves for each group^36^. Additionally, we applied the restricted cubic spline function (five knots) in Cox regression analyses to assess the possible nonlinear association between pre-diagnostic cruciferous vegetables intake and lung cancer-specific survival^37^.

In the initial analyses, we included all the patients and adjusted for demographic and lifestyle characteristics and other nonclinical prognostic factors that were related to both cruciferous vegetables intake and survival. These included age at diagnosis, body mass index (BMI), cigarette smoking, tea drinking, and intakes of total energy, fruit, and non-cruciferous vegetables. In the further analyses, we only included these patients (n = 331) with available data on clinical prognostic factors and further adjusted for TNM stage and cancer treatment (chemotherapy, radiotherapy, and surgery). Further inclusion of education level, physical activity, family history of lung cancer, history of lung disease (asthma, chronic bronchitis, and tuberculosis), alcohol drinking, soy food intake, use of non-steroidal anti-inflammatory drugs and antioxidant vitamin, time interval between the baseline dietary measurement and lung cancer diagnosis, and tumor histological type made no appreciable differences to the HR estimates and were not included in final multivariable models.

Three sensitivity analyses were conducted: (1) excluding 20 and 11 lung cancer cases who were diagnosed within the first year after baseline and the first follow-up FFQ to address the possibility that that women with underlying cancer may have altered their cruciferous vegetables intake, (2) only using the baseline FFQ to assess the dietary intake of the included patients, (3) and using cumulative averaging which reported by Meyerhardt *et al*^30^ to update the dietary exposures instead of using the average intakes estimated from the first FFQ (at baseline) and the second FFQ. For example, if a healthy woman completed first FFQ at 4 months, completed second FFQ at 28 months, and had a lung cancer at 40 months, the total time between first FFQ and lung cancer was 36 months and 66.7% of that time was between first FFQ and second FFQ and 33.3% of that time was between second FFQ and the lung cancer. We therefore calculated the cruciferous vegetables as follows: cumulative averaging cruciferous vegetables = (cruciferous vegetables at first FFQ × 0.667) + {[( cruciferous vegetables at first FFQ + cruciferous vegetables at second FFQ) / 2] × 0.333}. Likelihood ratio tests were also used to test for statistical interactions, which compared the model including only the main effects with the model including both main effects and interaction terms^38^. The validity of the proportional hazards assumption was tested by creating multiplicative interaction terms between cruciferous vegetables intake and survival time and comparing Cox models with and without interaction terms using the likelihood ratio test; no violations were observed. All reported *P* values are from two-sided tests. All statistical analyses utilized SAS software, version 9.3 (SAS Institute, Cary, NC).

## Additional Information

**How to cite this article**: Wu, Q.-J. *et al.* Pre-diagnostic cruciferous vegetables intake and lung cancer survival among Chinese women. *Sci. Rep.* **5**, 10306; doi: 10.1038/srep10306 (2015).

## Supplementary Material

Supplementary Information

## Figures and Tables

**Table 1:** Demographic and selected lifestyle characteristics and clinical predictors of lung cancer patients by quartile for pre-diagnostic cruciferous vegetables intake: the Shanghai Women’s Health Study, 1997 to 2011

**Characteristic**	**Cruciferous vegetables intake (quartiles, g/day)**				***P* value**
	**Q1 (< 53.2)**	**Q2 (53.2-83.3)**	**Q3 (83.4-118.6)**	**Q4 (≥ 118.7)**	
No. of Events	109	102	102	99	
Age at diagnosis (years), Mean (SD)	67.0 (9.0)	66.4 (9.1)	66.4 (9.4)	66.9 (8.6)	0.90
**Mean (SE)**†					
Time interval between first FFQ and cancer diagnosis (years)	7.2 (0.3)	7.6 (0.3)	7.6 (0.3)	7.3 (0.3)	0.54
Body mass index (kg/m^2^)	23.9 (0.3)	24.1 (0.3)	24.5 (0.3)	24.6 (0.3)	< 0.01
Physical activity (MET hour/week)	103.8 (4.1)	112.9 (4.0)	107.2 (4.0)	114.4 (4.0)	0.29
Total energy intake (kcal/day)	1431.8 (29.9)	1545.1 (29.8)	1642.1 (29.8)	1773.2 (29.8)	< 0.01
Fruit intake (g/day)	167.1 (11.8)	201.3 (11.7)	240.2 (11.7)	283.7 (11.7)	< 0.01
Non-cruciferous vegetables intake (g/day)	111.5 (9.2)	161.1 (9.2)	202.7 (9.2)	286.1 (9.2)	< 0.01
Soy food intake (g/day)	110.7 (9.5)	136.6 (9.5)	162.1 (9.5)	182.2 (9.5)	< 0.01
**No. (%)**‡					
Education level					0.44
Elementary school or less	66 (47.2)	48 (35.5)	55 (40.7)	60 (43.6)	
Middle school	30 (22.7)	38 (27.4)	33 (23.6)	33 (24.2)	
High school	22 (16.4)	31 (22.3)	38 (27.8)	21 (15.4)	
College or above	18 (13.6)	20 (14.8)	11 (7.9)	23 (16.8)	
Cigarette smoking	18 (13.2)	13 (9.7)	5 (3.7)	12 (8.7)	0.06
Tea drinking	42 (31.6)	33 (24.0)	41 (29.4)	48 (35.1)	0.24
History of lung disease§	32 (23.9)	29 (21.1)	24 (17.5)	25 (18.3)	0.60
Passive smoking¶	31 (25.1)	36 (29.6)	28 (21.6)	32 (25.6)	0.54
Tumor stage					0.31
I and II	10 (7.5)	8 (5.8)	15 (10.8)	12 (8.8)	
III	25 (18.7)	16 (11.7)	24 (17.2)	23 (16.8)	
IV	49 (35.6)	56 (40.4)	48 (35.5)	45 (32.9)	
Unknown	52 (38.3)	57 (42.1)	50 (36.5)	57 (41.5)	
Chemotherapy					0.73
Yes	78 (57.7)	86 (63.0)	84 (61.2)	87 (63.7)	
No	58 (42.3)	51 (37.0)	53 (38.8)	50 (36.3)	
Surgery					0.46
Yes	40 (30.0)	53 (38.9)	49 (35.1)	48 (35.2)	
No	96 (70.0)	84 (61.1)	88 (64.9)	89 (64.8)	
Radiotherapy					0.97
Yes	25 (18.8)	25 (18.1)	23 (16.5)	24 (17.6)	
No	111 (81.2)	112 (81.9)	114 (83.5)	113 (82.4)	
Histological type*					0.09
Nonadenocaricinoma	23 (34.5)	17 (22.0)	16 (16.2)	24 (27.0)	
Adenocarcinoma	43 (64.8)	64 (78.7)	73 (84.5)	65 (72.4)	

NOTE. For all characteristics except age at diagnosis, means (standard errors), or percentages were adjusted for age at baseline. Pre-diagnostic cruciferous vegetables intake was estimated by averaging the data from the first FFQ and the second FFQ surveys. For patients who provided no second FFQ data or reported having chronic diseases (e.g., diabetes, cardiovascular) or lung cancer diagnosed between the two FFQs, only the intake estimates from the first FFQ were used. Abbreviations: FFQ, food frequency questionnaire.

^†^Kruskal-Wallis test

^‡^Frequency; *χ*^2^ test

^§^Lung disease including asthma, chronic bronchitis, and tuberculosis.

^¶^Among non-smoking patients (n = 499).

^*^Among patients with available data on histological type (n = 325).

**Table 2:** Demographic and clinical characteristics and lung cancer specific survival among lung cancer patients with clinical information: the Shanghai Women’s Health Study, 1997 to 2011

**Characteristic**	**No./Events**	**HR**	**95% CI**†
Age at diagnosis (year)			
≤60	85/58	1.0	(Ref)
60-70	120/91	1.14	(0.81-1.61)
> 70	126/101	1.42	(1.00-2.03)
Overweight/obese (BMI ≥25 kg/m^2^)			
No	201/148	1.0	(Ref)
Yes	130/102	1.03	(0.79-1.33)
Education (High school and above)			
No	288/223	1.0	(Ref)
Yes	43/27	0.74	(0.49-1.11)
Cigarette smoking			
No	308/231	1.0	(Ref)
Yes	23/19	2.01	(1.23-3.30)
Tea drinking			
No	225/177	1.0	(Ref)
Yes	106/73	0.63	(0.47-0.83)
Tumor stage			
I and II	45/17	1.0	(Ref)
III	88/73	3.22	(1.84-5.64)
IV	198/160	5.13	(2.84-9.29)
Chemotherapy			
No	102/71	1.0	(Ref)
Yes	229/179	0.76	(0.57-1.02)
Surgery			
No	216/180	1.0	(Ref)
Yes	115/70	0.76	(0.54-1.07)
Radiotherapy			
No	263/196	1.0	(Ref)
Yes	68/54	0.84	(0.62-1.16)
Histological type‡			
Nonadenocaricinoma	45/34	1.0	(Ref)
Adenocarcinoma	171/116	0.94	(0.61-1.43)

^†^HRs (95% CIs) for lung cancer specific survival was estimated by using multivariable proportional hazards models, mutually adjusted for all other variables listed in the table except for pathologic type.

^‡^Data on the histological type of lung cancer were missing for 115 patients.

NOTE. Analyses were restricted to patients with lung cancer who had data available on both nonclinical and clinical variables (n = 331). Abbreviations: BMI, body mass index; CI, confidence interval; HR, hazards ratio.

**Table 3:** HR for lung cancer-specific survival among lung cancer patients according to pre-diagnostic cruciferous vegetables intake: the Shanghai Women’s Health Study, 1997 to 2011

**Cruciferous vegetables intake (g/day)**									
	Q1		Q2		Q3		Q4		*P*_trend_*
**All patients (N** = **547)**									
Range of intake (Median)	<53.2 (37.6)		53.2-83.4 (65.4)		83.4-118.7 (97.9)		≥118.7 (150.1)		
No./Events	136/100		137/100		137/99		137/94		
Model 1† HR (95% CI)	1.00	(Ref)	0.97	0.73-1.29	0.88	0.66-1.18	0.72	0.53-0.97	0.02
Model 2‡ HR (95% CI)	1.00	(Ref)	0.94	0.71-1.26	0.88	0.65-1.19	0.69	0.49-0.95	0.02
**Patients with data on clinical characteristics (N** = **331)**									
Range of intake (Median)	<52.1 (34.7)		52.1-84.4 (64.7)		84.4-116.8 (98.6)		≥116.8 (148.4)		
No./Events	82/60		83/66		83/65		83/59		
Model 1† HR (95% CI)	1.00	(Ref)	1.19	0.82-1.72	0.91	0.63-1.31	0.69	0.47-1.01	0.01
Model 3§ HR (95% CI)	1.00	(Ref)	0.95	0.65-1.39	0.91	0.62-1.34	0.63	0.41-0.97	0.03
**Patients who never smoked with data on clinical characteristics (N** = **308)**									
Range of intake (Median)	<53.1 (37.3)		53.1-85.5 (65.1)		85.5-116.4 (99.1)		≥116.4 (149.1)		
No./Events	77/56		77/62		77/59		77/54		
Model 1† HR (95% CI)	1.00	(Ref)	1.21	0.82-1.77	0.89	0.61-1.31	0.67	0.45-1.00	0.01
Model 4¶ HR (95% CI)	1.00	(Ref)	0.90	0.61-1.34	0.88	0.59-1.33	0.58	0.37-0.91	0.02

NOTE. Pre-diagnostic cruciferous vegetables intake was estimated by averaging the data from the first FFQ and the second FFQ surveys. For patients who provided no second FFQ data or reported having chronic diseases (e.g., diabetes, cardiovascular) or lung cancer diagnosed between the two FFQs, only the intake estimates from the first FFQ were used.Abbreviations: CI, confidence interval; HR, hazards ratio.

^*^P for trend; tests calculated by entering stratum-specific median values for cruciferous vegetables intake as continuous variables in Cox proportional hazards models.

^†^HRs (95% CIs) for lung cancer-specific survival was estimated by using multivariable proportional hazards models which were stratified on birth year and adjusted for adjusted for age at diagnosis and total energy intake.

^‡^Same as Model 1 and further adjusted for body mass index, tea drinking, cigarette smoking, intakes of fruit and non-cruciferous vegetables.

^§^Same as Model 2 and further adjusted for tumor stage, surgery, radiotherapy, and chemotherapy.

^¶^Same as Model 3 but without adjusting for cigarette smoking.
