# Supplementary material for: Pre-diagnostic cruciferous vegetables intake and lung cancer survival among Chinese women
Source: Sci Rep. 2015 May 19;5:10306. doi: 10.1038/srep10306 (PMC4650798; doi:10.1038/srep10306)
Supplement: Supplementary Information [file srep10306-s1.pdf]

Pre-diagnostic cruciferous vegetables intake and lung cancer survival among Chinese women

Qi-Jun Wu, Gong Yang, Wei Zheng, Hong-Lan Li, Jing Gao, Jing Wang, Yu-Tang Gao, Xiao-Ou Shu, Yong-Bing Xiang

Supplementary Table 1 HR for lung cancer-specific survival among lung cancer patients according to pre-diagnostic cruciferous vegetables intake after excluding cases that were diagnosed within the first year after baseline and the first follow-up food frequency questionnaire: the Shanghai Women's Health Study, 1997 to 2011

	Cruciferous vegetables intake (g/day)								
	Q1		Q2		Q3		Q4		P_{trend} *
**All patients (N = 516)**									
Range of intake (Median)	< 53.7 (38.6)		53.7-83.3 (66.0)		83.3-116.4 (97.5)		≥ 116.4 (148.4)		
No./Events	129/94		129/92		129/94		129/83		
Model 1 † HR (95% CI)	1.00	(Ref)	0.91	0.68-1.22	0.87	0.65-1.17	0.68	0.49-0.94	0.02
Model 2 ‡ HR (95% CI)	1.00	(Ref)	0.91	0.67-1.22	0.89	0.65-1.21	0.68	0.48-0.96	0.03
**Patients with data on clinical characteristics (N = 306)**									
Range of intake (Median)	< 52.4 (35.6)		52.4-83.2 (64.7)		83.2-110.3 (96.6)		≥ 110.3 (143.3)		
No./Events	76/55		77/60		76/58		77/53		
Model 1 † HR (95% CI)	1.00	(Ref)	1.09	0.74-1.60	0.85	0.58-1.24	0.71	0.47-1.07	0.04
Model 3 § HR (95% CI)	1.00	(Ref)	0.85	0.57-1.26	0.85	0.57-1.27	0.71	0.45-1.11	0.16
**Patients who never smoked with data on clinical characteristics (N = 284)**									
Range of intake (Median)	< 53.7 (37.5)		53.7-84.7 (65.1)		84.7-111.0 (96.7)		≥ 111.0 (146.0)		
No./Events	71/51		71/56		71/53		71/48		
Model 1 † HR (95% CI)	1.00	(Ref)	1.07	0.72-1.60	0.81	0.54-1.22	0.65	0.42-1.00	0.02
Model 4 ¶ HR (95% CI)	1.00	(Ref)	0.83	0.55-1.25	0.81	0.53-1.24	0.64	0.40-1.02	0.07

NOTE. Analyses were restricted to patients that excluded 20 and 11 lung cancer cases that were diagnosed within the first year after baseline and the first follow-up FFQ. Pre-diagnostic cruciferous vegetables intake was estimated by averaging the data from the first FFQ and the second FFQ

surveys. For patients who provided no second FFQ data or reported having chronic diseases (e.g., diabetes, cardiovascular) or lung cancer diagnosed between the two FFQs, only the intake estimates from the first FFQ were used.

Abbreviations: CI, confidence interval; HR, hazards ratio.

[*] *P* for trend; tests calculated by entering stratum-specific median values for cruciferous vegetables intake as continuous variables in Cox proportional hazards models.

[†] HRs (95% CIs) for lung cancer-specific survival was estimated by using multivariable proportional hazards models which were stratified on birth year and adjusted for adjusted for age at diagnosis and total energy intake.

[‡] Same as Model 1 and further adjusted for body mass index, tea drinking, cigarette smoking, intakes of fruit and non-cruciferous vegetables.

[§] Same as Model 2 and further adjusted for tumor stage, surgery, radiotherapy, and chemotherapy.

[¶] Same as Model 3 but without adjusting for cigarette smoking.

Supplementary Table 2 HR for lung cancer-specific survival among lung cancer patients according to pre-diagnostic cruciferous vegetables intake using cumulative averaging method: the Shanghai Women's Health Study, 1997 to 2011

	Cruciferous vegetables intake (g/day)								
	**Q1**		**Q2**		**Q3**		**Q4**		***P* trend** *
**All patients (N = 547)**									
Range of intake (Median)	< 51.8 (35.1)		51.8-82.4 (64.7)		82.4-117.7 (97.0)		≥ 117.7 (149.8)		
No./Events	136/101		137/100		137/95		137/97		
Model 1 † HR (95% CI)	1.00	(Ref)	0.95	0.71-1.27	0.84	0.63-1.13	0.74	0.55-1.00	0.03
Model 2 ‡ HR (95% CI)	1.00	(Ref)	0.94	0.70-1.26	0.86	0.63-1.16	0.71	0.52-0.99	0.03
**Patients with data on clinical characteristics (N = 331)**									
Range of intake (Median)	< 50.7 (34.2)		50.7-82.1 (64.1)		82.1-115.7 (97.2)		≥ 115.7 (147.6)		
No./Events	82/62		83/64		83/61		83/63		
Model 1 † HR (95% CI)	1.00	(Ref)	0.90	0.62-1.29	0.75	0.52-1.10	0.69	0.48-1.00	0.04
Model 3 § HR (95% CI)	1.00	(Ref)	0.79	0.54-1.15	0.89	0.60-1.32	0.66	0.44-1.01	0.10
**Patients who never smoked with data on clinical characteristics (N = 308)**									
Range of intake (Median)	< 51.9 (34.7)		51.9-83.0 (64.7)		83.0-116.6 (98.0)		≥ 116.6 (149.1)		
No./Events	77/57		77/61		77/55		77/58		
Model 1 † HR (95% CI)	1.00	(Ref)	1.02	0.70-1.50	0.77	0.52-1.14	0.71	0.48-1.05	0.04
Model 4 ¶ HR (95% CI)	1.00	(Ref)	0.79	0.53-1.18	0.89	0.59-1.35	0.62	0.40-0.97	0.06

NOTE. Analyses were using cumulative averaging reported by Meyerhardt et al [Ref. 16] to update the dietary exposures.

Abbreviations: CI, confidence interval; HR, hazards ratio.

* *P* for trend; tests calculated by entering stratum-specific median values for cruciferous vegetables intake as continuous variables in Cox

proportional hazards models.

† HRs (95% CIs) for lung cancer-specific survival was estimated by using multivariable proportional hazards models which were stratified on birth year and adjusted for adjusted for age at diagnosis and total energy intake.

‡ Same as Model 1 and further adjusted for body mass index, tea drinking, cigarette smoking, intakes of fruit and non-cruciferous vegetables.

§ Same as Model 2 and further adjusted for tumor stage, surgery, radiotherapy, and chemotherapy.

¶ Same as Model 3 but without adjusting for cigarette smoking.

Supplementary Table 3 HR for lung cancer-specific survival among lung cancer patients according to pre-diagnostic cruciferous vegetables intake only using baseline food frequency questionnaire: the Shanghai Women's Health Study, 1997 to 2011

	Cruciferous vegetables intake (g/day)								
	Q1		Q2		Q3		Q4		P_{trend} *
**All patients (N = 547)**									
Range of intake (Median)	< 46.2 (27.1)		46.2-77.1 (58.2)		77.1-119.7 (90.8)		≥ 119.7 (154.3)		
No./Events	136/95		137/96		137/105		137/97		
Model 1 † HR (95% CI)	1.00	(Ref)	0.85	0.64-1.13	1.00	0.75-1.33	0.77	0.57-1.04	0.15
Model 2 ‡ HR (95% CI)	1.00	(Ref)	0.82	0.61-1.10	0.97	0.72-1.30	0.72	0.53-1.00	0.09
**Patients with data on clinical characteristics (N = 331)**									
Range of intake (Median)	< 45.9 (28.1)		45.9-75.7 (58.1)		75.7-120.5 (91.1)		≥ 120.5 (153.1)		
No./Events	82/58		83/64		83/65		83/63		
Model 1 † HR (95% CI)	1.00	(Ref)	0.91	0.63-1.31	0.90	0.62-1.29	0.70	0.48-1.02	0.06
Model 3 § HR (95% CI)	1.00	(Ref)	0.89	0.62-1.29	0.96	0.66-1.39	0.68	0.45-1.02	0.06
**Patients who never smoked with data on clinical characteristics (N = 308)**									
Range of intake (Median)	< 46.9 (29.2)		46.9-76.4 (58.6)		76.4-123.3 (92.6)		≥ 123.3 (160.0)		
No./Events	77/55		77/59		77/59		77/58		
Model 1 † HR (95% CI)	1.00	(Ref)	0.92	0.63-1.34	0.88	0.60-1.28	0.69	0.47-1.01	0.04
Model 4 ¶ HR (95% CI)	1.00	(Ref)	0.94	0.64-1.38	0.98	0.67-1.45	0.67	0.44-1.02	0.04

NOTE. Analyses were only using the first food frequency questionnaire (at baseline) to assess the dietary exposures.

Abbreviations: CI, confidence interval; HR, hazards ratio.

* *P* for trend; tests calculated by entering stratum-specific median values for cruciferous vegetables intake as continuous variables in Cox

proportional hazards models.

† HRs (95% CIs) for lung cancer-specific survival was estimated by using multivariable proportional hazards models which were stratified on birth year and adjusted for adjusted for age at diagnosis and total energy intake.

‡ Same as Model 1 and further adjusted for body mass index, tea drinking, cigarette smoking, intakes of fruit and non-cruciferous vegetables.

§ Same as Model 2 and further adjusted for tumor stage, surgery, radiotherapy, and chemotherapy.

¶ Same as Model 3 but without adjusting for cigarette smoking.
